# Toward an Optimal Global Stem Cell Donor Recruitment Strategy

**DOI:** 10.1371/journal.pone.0086605

**Published:** 2014-01-30

**Authors:** Alexander H. Schmidt, Jürgen Sauter, Julia Pingel, Gerhard Ehninger

**Affiliations:** 1 DKMS German Bone Marrow Donor Center, Tübingen, Germany; 2 Internal Medicine I, University Hospital Carl Gustav Carus, Dresden, Germany; University of Pécs Medical School, Hungary

## Abstract

Population-specific matching probabilities (MP) are a key parameter to assess the benefits of unrelated stem cell donor registries and the need for further donor recruitment efforts. In this study, we describe a general framework for MP estimations of specific and mixed patient populations under consideration of international stem cell donor exchange. Calculations were based on population-specific 4-locus (HLA-A, -B, -C, -DRB1) high-resolution haplotype frequencies (HF) of up to 21 populations. In various scenarios, we calculated several quantities of high practical relevance, including the maximal MP that can be reached by recruiting a fixed number of donors, the corresponding optimal composition by population of new registrants, and the minimal number of donors who need to be recruited to reach a defined MP. Starting at current donor numbers, the largest MP increases due to *n* = 500,000 additional same-population donors were observed for patients from Bosnia-Herzegovina (+0.25), Greece (+0.21) and Romania (+0.20). Especially small MP increases occurred for European Americans (+0.004), Germans (+0.01) and Hispanic Americans (+0.01). Due to the large Chinese population, the optimal distribution of *n* = 5,000,000 new donors worldwide included 3.9 million Chinese donors. As a general result of our calculations, we observed a need for same-population donor recruitment in order to increase population-specific MP efficiently. This result was robust despite limitations of our input data, including the use of HF derived from relatively small samples ranging from *n* = 1028 (Bosnia-Herzegovina) to *n* = 33,083 (Turkey) individuals. National strategies that neglect domestic donor recruitment should therefore be critically re-assessed, especially if only few donors have been recruited so far.

## Introduction

Allogeneic hematopoietic stem cell transplantation (HSCT) is an increasingly used therapy for leukemia and other severe blood diseases [1], [2]. Unrelated donor searches are carried out for patients without human leukocyte antigen (HLA)-identical siblings. Due to the extensive diversity of the HLA system and the need for exact donor-recipient HLA matching, large unrelated donor registries have been established. As of June 2013, there were more than 21.1 million potential stem cell donors in 69 registries from 50 countries [3]. 14,209 unrelated stem cell donations were reported to the World Marrow Donor Association in 2011. Of these donations, 6,513 (45.8%) were cross-border [4].

The probability to find at least one HLA-matched donor for a patient from a specific population in a defined donor file is a key parameter in strategic donor registry planning. The definition of an HLA-matched donor has changed over time. Currently, a donor who matches with a patient at least for the HLA loci A, B, C and DRB1 at high resolution (“8/8 high-resolution matching”) is regarded as HLA-matched [5]–[9]. For high-resolution typing, all alleles are resolved within the antigen recognition site. This requires sequencing of exons 2 and 3 for class I genes (HLA-A, -B, and -C) and exon 2 for HLA-DRB1. Contrary to the one-step sequencing strategy proposed by Tu et al. [10], cis-trans ambiguities within the relevant exons are resolved while synonymous mutations inside or outside the relevant exons are not taken into account. Allele ambiguities from non-synonymous mutations outside the relevant exons are also not resolved.

Methods for the calculation of matching probabilities (MP) by donor file size have been developed since the 1980s [11], [12] and are well established [13]–[15]. They are based on population-specific HLA haplotype frequencies (HF) that can be estimated from phenotypic data using appropriate tools as the expectation-maximization algorithm [16], [17].

Many MP analyses restrict to one or two populations. i.e., they consider one donor population and one patient population that may be identical or not. Given the considerable amount of international exchange of stem cell products, these models seem to be oversimplifying. MP for specific patient populations that consider donors from various populations have been derived by Dehn et al. [18]. Due to the empirical nature of that study, an extrapolation of the results to larger donor file sizes is difficult. In a previous study, we used simulation techniques to estimate MP of Polish patients for varying sizes of a combined Polish-German donor file [19]. Here, we describe a general framework for MP estimations of specific or combined patient populations in a model including international stem cell donor exchange. We apply our framework to real data for up to 21 populations. Practical applications of this approach have come into reach as population-specific high-resolution 4-locus HF are increasingly available.

## Methods

### Calculation of Matching Probabilities

The probability *p(n)* for a random patient from a given population to find at least one matching donor in a registry including *n* individuals of a donor population is given by 
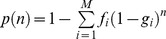
 with *f_i_* and *g_i_* being the frequencies of the *i*-th phenotype in the patient and donor populations, respectively. Here and in the following, *M* is the number of different phenotypes. Phenotype frequencies are derived from known HF under the assumption of Hardy-Weinberg equilibrium (HWE). Throughout this paper, we analyze 8/8 high-resolution matching. The phenotypes considered are, therefore, HLA-A, -B, -C, -DRB1 high-resolution phenotypes. All methods used would also be applicable to donor-recipient matching based on different genes or typing resolutions.

It has been shown [20] that the probability *p_k_* to find at least one matching donor for a patient of population *k* in a registry including donors from *N* populations is given by 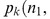 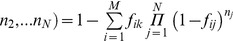 where *n_j_* indicates the number of donors from population *j* in the registry and the frequency of the *i*-th phenotype in population *j* is denoted by *f_ij_*. The probability *p_k_* describes a scalar field over the *N*-dimensional space *U_N_* of population-specific donor numbers. Mathematical properties of this scalar field are described in File S1.

In order to assess global benefits of a donor registry, it is also necessary to estimate MP for a combined patient population. If *m_k_* describes the fraction of patients of population *k* with donor searches in a defined time interval then 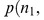 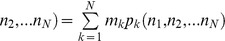 describes the MP for the combined patient population. For our example calculations, we assumed the numbers of patients with donor searches were proportional to population sizes.

### Optimization of Matching Probabilities

We analyzed two optimization scenarios: First, we determined the optimal composition of new donors and resulting maximum values of population-specific and combined MP under the assumption that a fixed number of new donors is added to a donor registry characterized by population-specific donor numbers and HF. Second, we determined minimum numbers of new donors who need to be added to the current registry in order to increase certain MP to defined targets as, for example, *p_k_* = 0.8. Mathematical details of the algorithm are given in the File S2.

### Input Data for Model Calculations

All HF data processed in this work were calculated from samples with size *n*≥1,000 of registered stem cell donors. Polish and German HF were based on *n* = 20,653 donors of DKMS Polska [19] and *n* = 8,862 donors of DKMS Germany [15], respectively. HF data for the four American populations were derived from donors of the National Marrow Donor Program of the Unites States [21], [22]. Sample sizes for these populations ranged from *n* = 1,750 (Asian/Pacific Americans) to *n* = 7,867 (European Americans). More detailed data regarding US populations [23] were not publicly available at the time of data analysis. The remaining 15 population-specific HF were estimated from minority donors of DKMS Germany [24]. All four sources of HF data [15], [19], [22], [24] tested for deviations from HWE. While some statistically significant deviations from HWE could be identified in three of these studies, their impact on HF estimations was expected to be small.

For each population, we included HF *h_i_* up to a cumulated frequency of 0.995 with 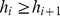. Phenotype frequencies that were entered into the model calculations were derived from HF under the assumption of HWE.

Numbers of registered donors were taken primarily from Bone Marrow Donors Worldwide [3] as of February 8, 2012 where available. For the four different American populations, we used figures published on the website of the National Marrow Donor Program of the United States [21]. We then added DKMS minority donors to the respective populations according to their self-assessments and removed them from the German donor file. For the populations “Bosnia-Herzegovina”, “Romania”, and “Kazakhstan”, no donors were included in the BMDW data. Thus, for these populations donor numbers are only due to DKMS minority donors. Donor numbers that were obtained this way are displayed in Table 1. The number of donors was not limited by completeness and resolution of their respective HLA typing as given by the registries, i.e., we did not restrict to donors with certain minimum typing requirements.

**Table 1:** Overview on populations included in the analyses.

	Sample size	# of donors	Population size (in millions)
African American	2,386	787,081	37.9
Asian/Pacific American	1,750	769,846	14.6
Austria	1,698	66,014	8.4
Bosnia-Herzegovina	1,028	1,754	3.8
China	1,282	403,945	1,336.7
Croatia	2,057	30,823	4.4
European American	7,867	3,690,624	196.9
France	1,406	200,172	65.0
Germany	8,862	4,343,558	81.5
Greece	1,894	37,760	11.3
Hispanic American	1,973	1,080,082	50.7
Italy	4,972	345,265	60.0
Kazakhstan	1,676	2,833	15.6
Poland	20,653	297,464	38.1
Portugal	1,176	283,523	10.6
Romania	1,234	2,421	21.5
Russia	4,621	16,200	142.9
Spain	1,107	93,623	47.2
The Netherlands	1,374	42,733	16.6
Turkey	33,083	114,248	73.7
United Kingdom	1,043	815,660	62.7

Population sizes as of 2011 were taken from the CIA World Factbook [25] for all populations associated with a country. For the four American populations, we used 2010 figures of the US Bureau of census [26]. Table 1 lists the population sizes used for analyses.

## Results

We analyzed two sets of populations using our model: The first set included only two different populations (Spanish and German) and was primarily aimed at demonstrating general principles of the analyses. The second example included 21 different populations (see Table 1) and served as first approximation to a real-world scenario. For each example, we discuss the optimal number and composition of new donors with respect to an increase of MP for patients from specific and combined populations.

### Calculations for Two Populations

**Figure 1:**
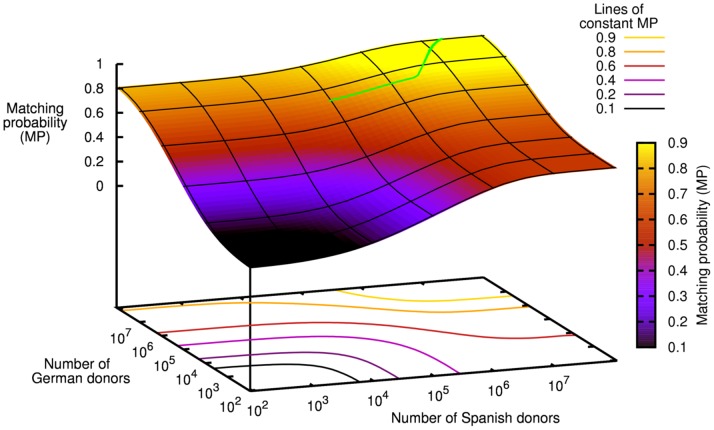
MP by registry size and composition for a combined patient population from a registry including Spanish and German donors. The green line indicates the optimal path of donor recruitment.

We calculated MP for Spanish and German patients (Figure S3 displayed in File S3, ) and for a combined patient population (Figure 1) as a function of registry size and composition. For the current donor numbers (*n*_Spanish_ = 93,623, *n*_German_ = 4,343,558), we obtained: *p*_Spanish_ = 0.57, *p*_German_ = 0.83, *p*_combined_ = 0.74. Without donors from the respective other population, population-specific MP reduced to *p*_Spanish_(*n*_Spanish_ = 93,623, *n*_German_ = 0) = 0.45 and *p*_German_(*n*_Spanish_ = 0, *n*_German_ = 4,343,558) = 0.86. It follows that Spanish patients currently benefit from German donors while German patients do not benefit significantly from Spanish donors. Without consideration of the respective own population, we obtained *p*_Spanish_(*n*_Spanish_ = 0, *n*_German_ = 4,343,558) = 0.41 and *p*_German_(*n*_Spanish_ = 93,623, *n*_German_ = 0) = 0.22. The fact that the calculated MP for Spanish patients from a relatively small file including only Spanish donors is slightly higher than from a German file of 46-fold size suggests a high relevance of domestic donor recruitment even when these two European populations are considered.

When 500,000 Spanish donors were added to the current registry, the updated MP were *p*_Spanish_ = 0.77, *p*_German_ = 0.83, and *p*_combined_ = 0.80. The addition of 500,000 German donors, on the other hand, led to the following MP: *p*_Spanish_ = 0.56, *p*_German_ = 0.84, *p*_combined_ = 0.74. Though existing German donors substantially contributed to current MP of Spanish patients (*p* = 0.57 with German donors, *p* = 0.45 without German donors), the addition of 500,000 German donors did not further improve MP of Spanish patients substantially. Generally, MP for Spanish or German patients were most efficiently increased if only Spanish or German donors were recruited, respectively.

**Figure 2:**
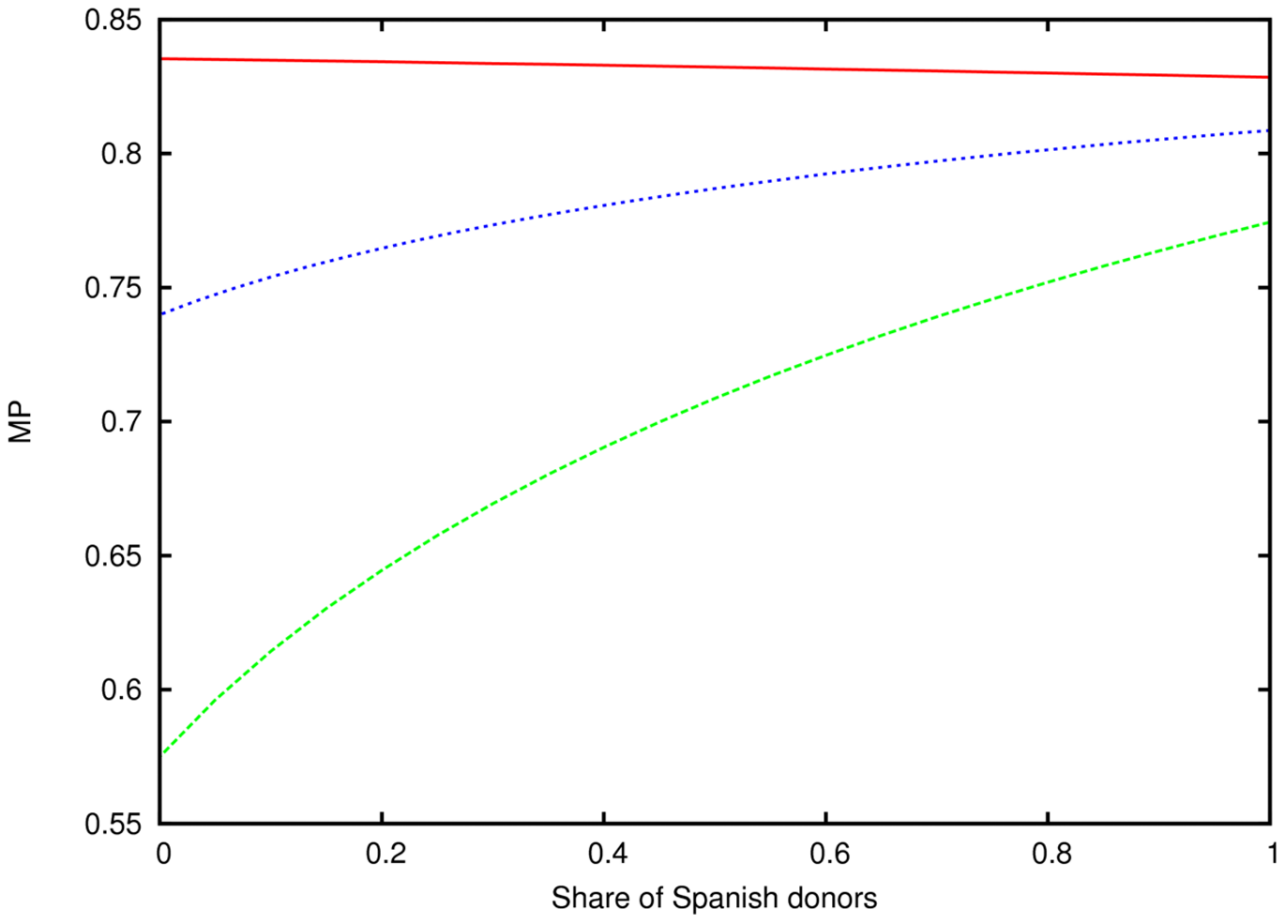
Matching probabilities for various patient populations (green: Spanish, red: German, blue: combined) by the share of Spanish donors among *n* = 500,000 new donors.

With respect to MP of the combined patient population, it was most efficient to only recruit Spanish donors when less than 2.3 million donors were added to the registry. Figure 2 shows, for the case of 500,000 new donors, MP changes by the fraction of Spanish donors. For larger numbers of new registrants, the optimal mix included both Spanish and German donors. For 3,000,000 (5,000,000) new donors, for example, it is optimal to recruit 87.8% (65.1%) Spanish and 12.2% (34.9%) German donors resulting in *p*_combined_ = 0.87 (*p*_combined_ = 0.89).

### Calculations for 21 Populations

**Figure 3:**
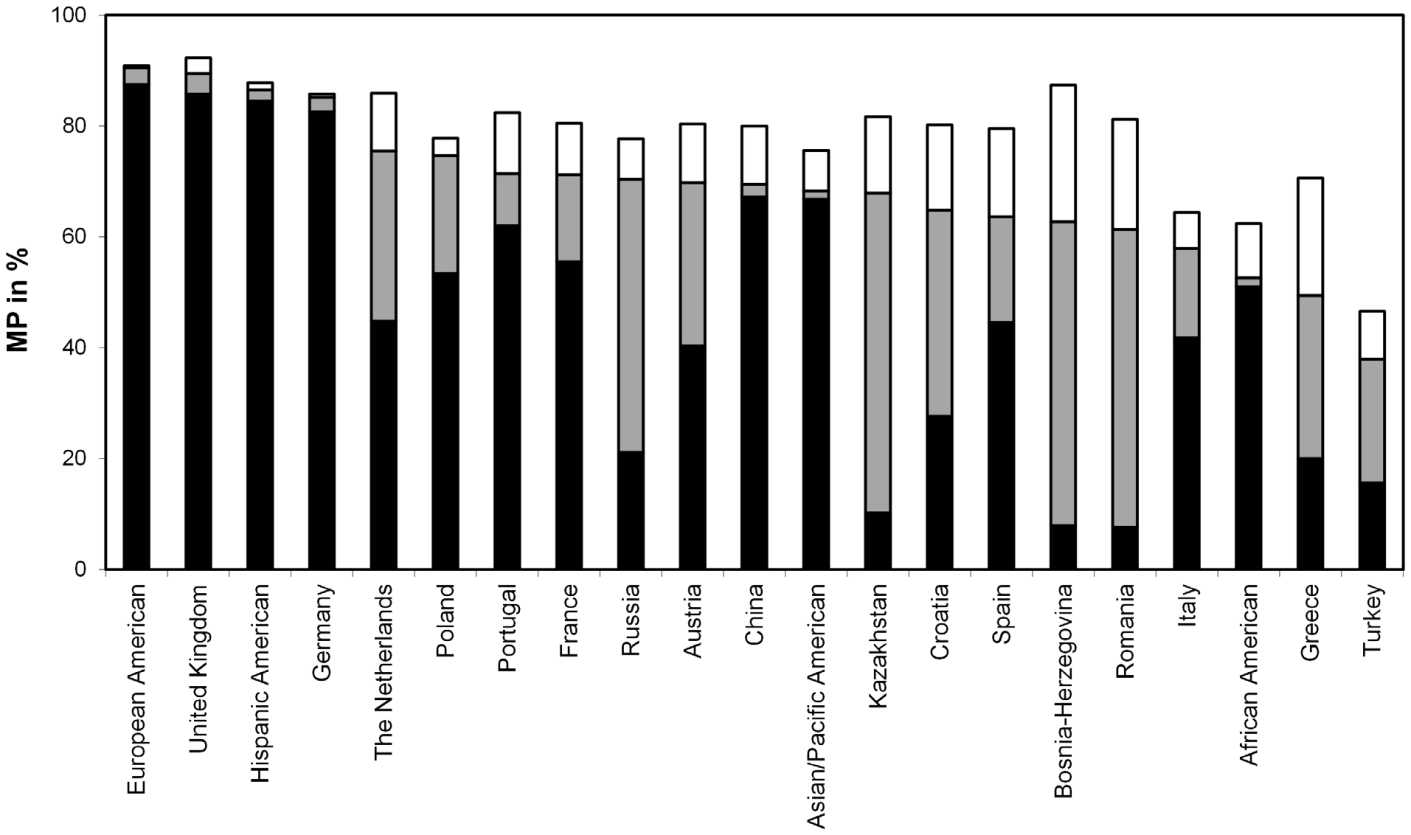
Population-specific MP from donors of the same population (black, donor numbers from *n* = 1,754 (Bosnia-Herzegovina) to *n* = 4,343,558 (Germany)) and increments from donors of other populations (grey) and from 500,000 additional donors of the same population (white). Populations are ordered by decreasing MP from the complete current registry, represented by the addition of black and grey columns.

We estimated MP for 21 populations and analyzed population-specific MP changes induced by the recruitment of 500,000 donors from the same population (Figure 3). Population-specific MP from same-population donors ranged from 0.08 for Romania to 0.88 for European Americans. For obvious reasons, these MP corresponded strongly with donor numbers by population (Spearman’s rank correlation coefficient, ρ = 0.90). For 4 populations, donor numbers and MP ranks differed by more than 3. MP ranks were lower than donor number ranks for African Americans (ranks 10 and 5, respectively), Italy (ranks 13 and 8) and Turkey (ranks 18 and 12), thus suggesting an especially high intra-population diversity for these populations. The opposite observation was made for the Netherlands (ranks 11 and 15, respectively).

As most populations and the majority of donors included in the model are of European origin, it is not surprising that non-European populations had the smallest increase in their MP due to donors from other populations, namely Asian/Pacific Americans (+0.02), African Americans (+0.02), Hispanic Americans (+0.02) and China (+0.02). The following populations with large donor numbers also showed small MP increases from donors of other populations: Germany (+0.03), European Americans (+0.03) and United Kingdom (+0.04). On the other hand, populations with small donor numbers such as Kazakhstan (+0.58), Bosnia-Herzegovina (+0.55) and Romania (+0.54) benefited most from international donors. As expected, MP that were calculated under consideration of all current donors were less strongly correlated to population-specific donor numbers (Spearman’s rank correlation coefficient, ρ = 0.503).

The largest MP increase by the recruitment of 500,000 new donors from the same population was obtained for Bosnia-Herzegovina (+0.25) followed by Greece (+0.21) and Romania (+0.20). Especially small MP increases were observed for European Americans (+0.004), Germany (+0.006) and Hispanic Americans (+0.01). These populations were among the four populations with the highest MP from the total current registry. For these four populations, the current MP was already larger than 0.8. For the other 17 populations, the number of same-population donors needed to reach 0.8 MP ranged from *n* = 124,829 for the Netherlands to *n* = 18,425,025 for Turkey (see Figure S4 in File S4, ).

We also calculated the optimal composition of *n* = 5,000,000 new donors in order to increase the MP of the combined patient population most efficiently. Due to the very large Chinese population, the result indicated a strong need for additional Chinese donors. The optimal composition of new donors included more than 3.88 million Chinese donors (77.7% of all new donors). Complete results are shown in File S5.

**Figure 4:**
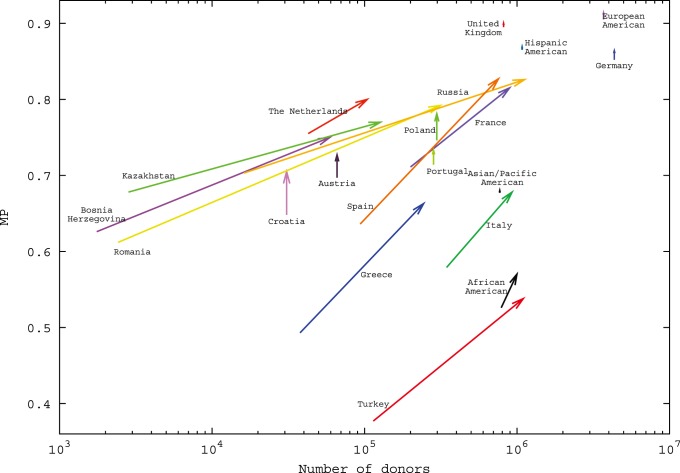
Changes in donor numbers and population-specific MP induced by optimal recruitment of n = 5,000,000 donors. Chinese donors and patients were not included in the analysis.

In order to get a more detailed picture of the 20 non-Chinese populations, we also carried out calculations without Chinese donors and patients. Results are displayed in Figure 4. Complete figures are given in File S6. The optimal composition of *n* = 5,000,000 new donors included donors from 11 populations, with numbers ranging from *n* = 57,472 (Bosnia-Herzegovina) to *n* = 1,103,536 (Russia). In that optimal scenario, the smallest MP increases were observed for Asian/Pacific Americans (+0.003), Hispanic Americans (+0.004) and United Kingdom (+0.01). Spain (+0.19), Romania (+0.18) and Greece (+0.17) had the largest MP increases. The MP of the combined patient population increased from 0.73 to 0.80. As we optimized the MP for a combined patient population, it is not surprising that the optimal composition of new donors did not include individuals from all 20 populations. Apart from population-specific HF and genetic relatedness between various populations, population sizes and population-specific donor numbers are main factors influencing the optimal composition of new donors. It is, therefore, consistent that the optimal composition included, for example, 1.1 million new Russian (population size: 142.9 million, registered donors: 16,200) but no German (population size: 81.5 million, registered donors: 4.3 million) donors.

Patients from populations with new donors in the optimal recruitment scenario benefitted most from recruitment of same-population donors. The MP for Spanish patients, for example, increased from *p*_Spanish_ = 0.64 to *p*_Spanish_ = 0.83. If only the *n* = 662,283 new Spanish donors in the optimal recruitment scenario were added to the original registry, the MP increased to *p*_Spanish_ = 0.82. The addition of the n = 4,337,717 non-Spanish donors to the original registry, on the other hand, resulted in *p*_Spanish_ = 0.66, thus again suggesting a need for same-population donor recruitment.

### Sample Size Effects

**Figure 5:**
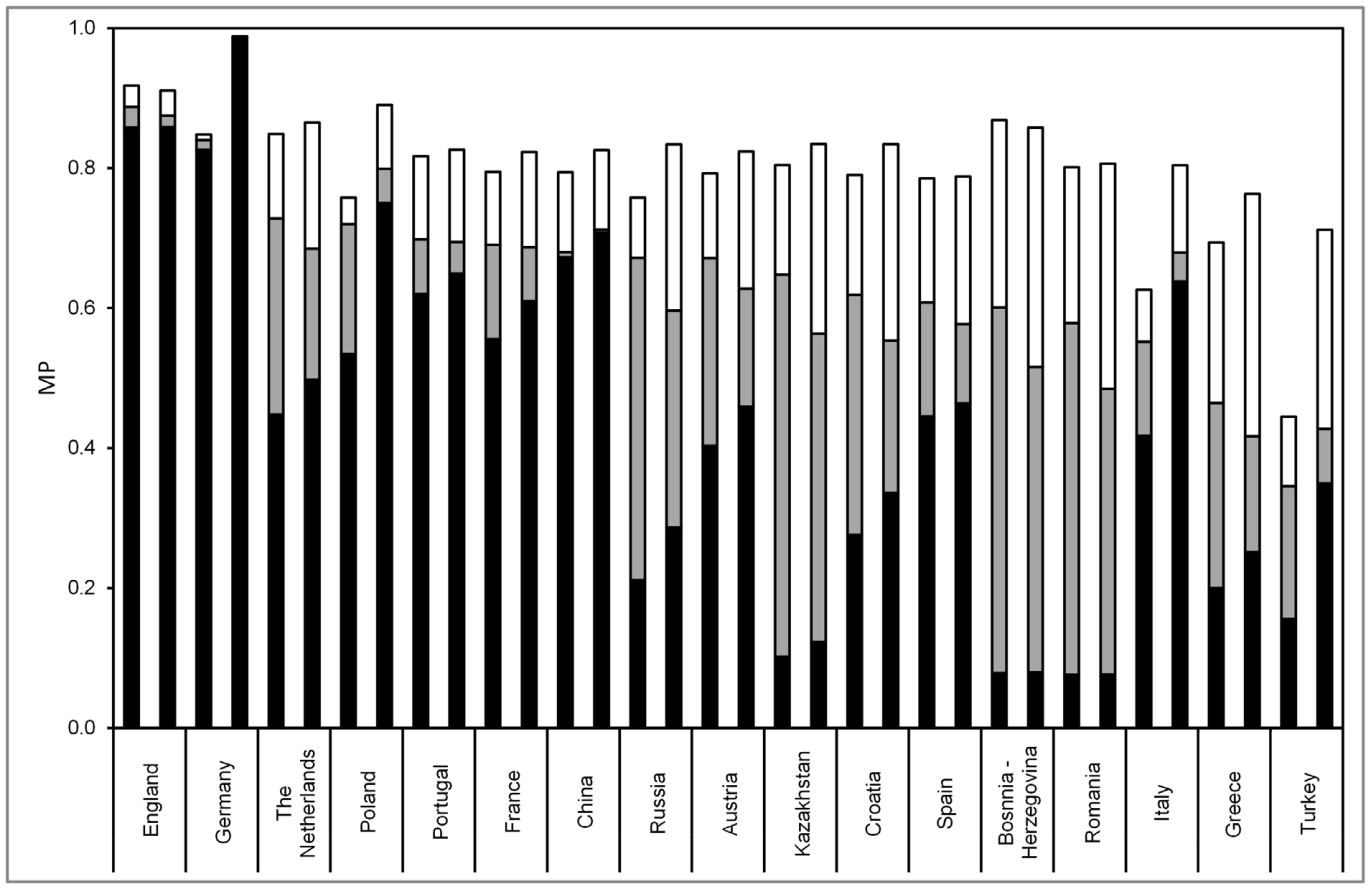
Population-specific MP from donors of the same population (black) and increments from donors of other populations (grey) and from 500,000 additional donors of the same population (white) in a model with 17 populations with available raw data. Scenario 1 (first column for each population) was based on the sample sizes given in Table 1. Scenario 2 was based on random sub-samples of size n = 1000. Populations are ordered by decreasing MP from the complete current registry, represented by the addition of black and grey columns, in Scenario 1.

We analyzed 17 populations with available raw data based on HF derived from two different sample sets: complete samples as given in Table 1 (scenario 1) and random sub-samples with size *n* = 1000 (scenario 2). Results are displayed in Figure 5.

For all populations, the calculated MP from same-population donors (black columns in Figure 5) were higher in scenario 2, thus reflecting the less complete representation of intra-population diversity by smaller samples. MP differences ranged from 0.0004 (Romania) to 0.22 (Italy) and were, as expectable, strongly correlated with sample size differences between both scenarios (Spearman’s rank correlation coefficient, ρ = 0.919). In spite of the considerable variation of between-scenario MP differences, MP from same-population donors were highly correlated between both scenarios (Spearman’s rank correlation coefficient, ρ = 0.951).

The benefits from international donor exchange (i.e., MP increase by donors from the international registry; grey columns in Figure 5) were higher in Scenario 1 for all populations. This observation results from two factors: overestimation of MP from same-population donors in scenario 2, and larger “overlap” between populations when existing intra-population diversity is better captured by larger samples. The benefits from international donor exchange were strongly correlated between both scenarios (Spearman’s rank correlation coefficient, ρ = 0.983).

Regarding MP including international donor exchange (sum of black and grey columns in Figure 5), the combination of the abovementioned sample size effects on MP from same-population donors and on MP increase from the international registry resulted in an MP increase for 5 populations (including Turkey, Poland, Germany and Italy, the four populations with the largest sample sizes) and an MP decrease for 12 populations in Scenario 2 compared to Scenario 1. MP including international donor exchange were well correlated between both scenarios (Spearman’s ρ = 0.885) with Italy showing the strongest discrepancy between both scenarios (rank #15 in scenario 1, rank #8 in scenario 2).

Benefits of ongoing domestic recruitment efforts (i.e., MP increase by 500,000 additional donors from the same population; white columns in Figure 5) were higher in Scenario 2 for all populations apart from Germany and China with MP differences ranging from −0.005 (Germany) to 0.19 (Turkey). Benefits of ongoing recruitment were well correlated between both scenarios (Spearman’s rank correlation coefficient, ρ = 0.814). The largest discrepancy could be observed for Turkey (rank #12 in scenario 1, rank #4 in scenario 2).

**Figure 6:**
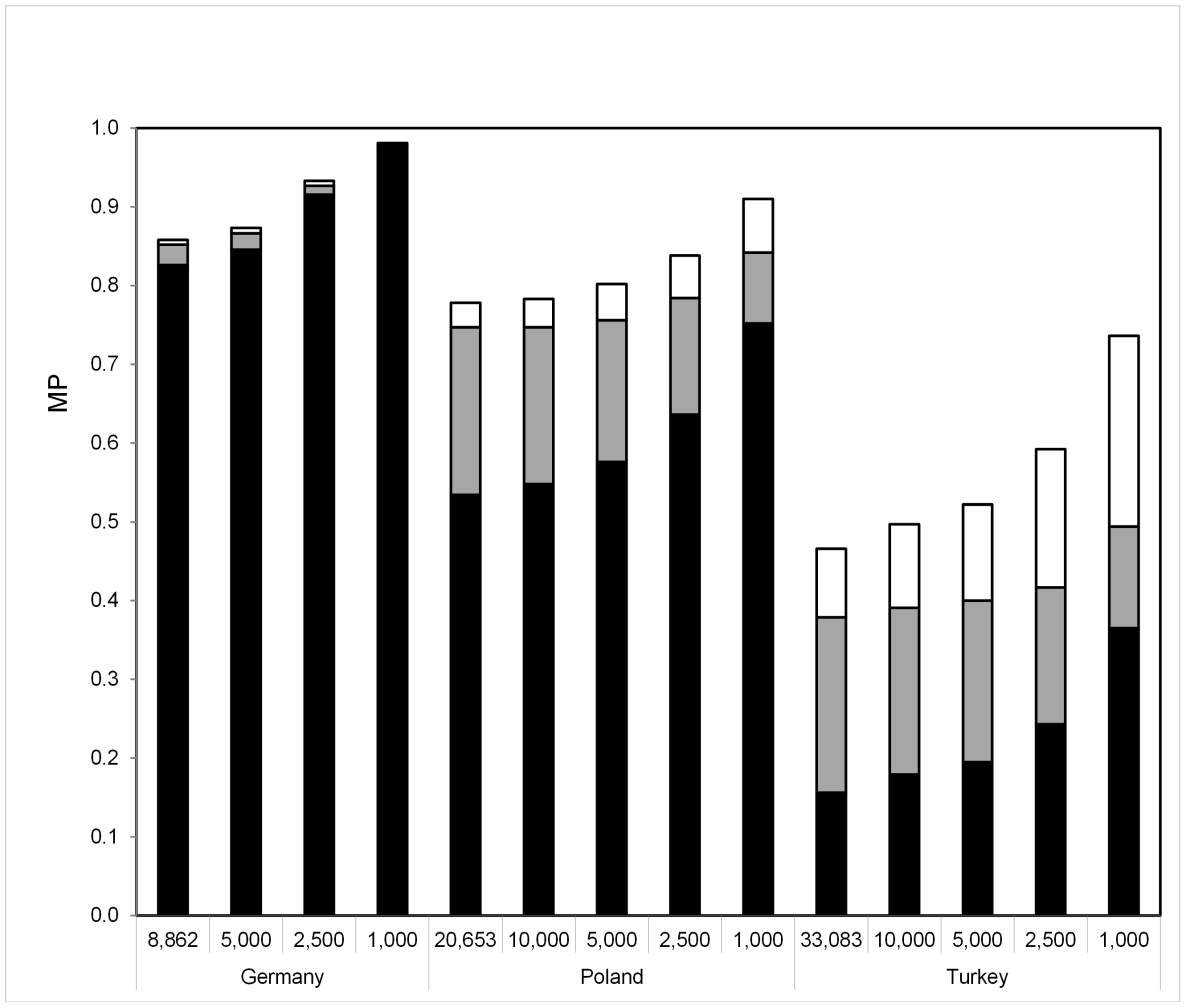
Population-specific MP for German, Polish and Turkish patients based on HF derived from various sample sizes as shown at the x-axis. Black, grey and white columns are defined as in Figure 3.

Figure 6 shows how MP for patients from Turkey, Poland and Germany changed when population-specific HF were derived from smaller sample sizes of *n* = 10,000 (analyses for Turkey and Poland only), *n* = 5,000, *n* = 2,500 and *n* = 1,000. For each analysis including 21 populations, all sample sizes apart from the analyzed one remained as given in Table 1.

MP for Polish patients, for example, increased only slightly when the sample size decreased from *n* = 20,653 to *n* = 5,000: from 0.53 to 0.58 for domestic donors only, from 0.75 to 0.76 for the total donor file including donors from 21 populations, and from 0.78 to 0.80 with 500,000 additional domestic donors. For smaller samples, however, MP for Polish patients were substantially affected by sample size. MP were 0.75, 0.84 and 0.91 in the three respective scenarios with sample size *n* = 1,000. MP for German and Turkish patients showed a similar pattern, thus suggesting an only modest dependency of MP from sample size for *n*≥5,000 while smaller sample sizes showed more relevant impact on calculated MP values.

## Discussion

In this work, we extended established methods for the estimation of population-specific MP to analyze scenarios including international stem cell donor exchange. We applied our framework to real HF data of up to 21 populations.

As a general result, we consistently observed considerable benefits of intra-population donor recruitment. This is surprising due to the large international registry of more than 21.1 million registered donors and the current level of international exchange (45.8% of all unrelated donations in 2011; [4]).

Our model was based on the assumption that donors who are full HLA matches to patients in need of a transplant will always be identified in the donor search process. There is, however, evidence that fully matching donors may remain unidentified in the donor search process due to incomplete HLA information [27]–[29]. Possible restrictions of international donor exchange due to socio-economic, political or other factors were also not considered in our model.

It needs to be emphasized that the model used in this work is focused strictly on patient benefits that were operationalized by the probability to find at least one HLA-matched donor in the global registry for a patient in need of a transplant. The model does not provide any financial cost-benefit estimates of donor recruitment activities carried out in specific countries and/or targeted at defined populations. Among other things, population-specific donor recruitment costs would be required for that purpose.

The nature of our input data led to limitations of our analyses, including: First, the study included about 13 million donors that represent only about 2/3 of the worldwide donor pool.

Second, HF data for 15 of 21 populations included in our calculations were estimated from German minority donors. Though we have previously shown that such HF correspond satisfyingly with HF derived from samples from the respective countries [23], [30], this approach is not optimal for several reasons, including: Immigrants to Germany build no random samples of their respective home populations, no information on the regions of origin in the respective home countries is available, and immigrants’ self-assessments of ethnic descent may have substantial error rates that are difficult to quantify.

Third, the optimal definition of populations that enter the analysis is not self-evident. Regional HLA frequency differences have been described for several countries [31]–[35]. Nevertheless, it may not be useful to define populations too narrow as corresponding sample sizes will decrease and related problems will aggravate. On the other hand, the country-based definition of populations as used for the 17 non-US populations in our calculations may be oversimplifying at least for countries with heterogeneous population structure as, for example, China [36], [37] or Turkey. Further, the four US populations are not well-defined. For instance, it has been shown [38] that Eastern European American donors genetically differ from Western European American donors.

Fourth, we used the numbers of registered donors by population for modeling the current registry, thus neglecting issues of donor availability. In principle, this problem can easily be resolved by introducing a factor describing donor availability. As donor availability rates differ between various populations [39], this factor is required to be population-specific. In our setting, it has not been included as availability data based on a sufficient number of typing requests were not available for all populations.

Fifth, calculations regarding MP of combined patient populations were based on the assumption that patients from all populations have same access to unrelated HSCT when MP issues are not considered. It is, however, known that, in practice, the number of patients from a specific population for whom a donor is searched depends on various factors including population size, population-specific disease incidences, and socioeconomic parameters [40].

Sixth, most HF used for calculations were based on samples of sizes between *n* = 1000 and *n* = 5000 while our results suggest that estimated MP values may strongly deviate from “real” values for *n*<5000. Future studies should nevertheless aim at a larger and more homogeneous input data for HF estimate with respect to sample sizes. Further, there is evidence that expectation-maximization algorithms which are used to compute HF do not perform well not only when the input sample size is small but also in cases of high polymorphism (as for HLA) and when distant loci (such as, for example, HLA-A and HLA-DRB1) are included [41].

Seventh, we assumed that donor and patient HF are identical for each population, thus neglecting potential HLA associations with diseases that may be treated by stem cell transplantation.

Eighth, of the 84 population-locus combinations that entered the analyses of our study, 12 showed deviations from HWE that were statistically significant [15], [19], [22], [24]. In 10 of these cases, deviations from HWE were associated with excess homozygosity. It has been shown that deviations from HWE do not affect the accuracy of HF estimations with the EM algorithm negatively in these cases [42]. In one case (HLA-DR for Poland), the *p* value of the test for deviation from HWE was 0.011, thus not indicating a quantitatively relevant deviation from HWE [13]. The remaining combination with significant deviation from HWE (HLA-A for European American) showed both a small *p* value (<10^−4^) and excess heterozygosity. Though the relatively large size of the underlying sample (*n* = 7,867) may have contributed to the small *p* value, this deviation from HWE creates a potential source of error.

Ninth, new HLA alleles are identified at an increasing rate [43], [44]. Carriers of yet unassigned alleles were excluded from HF estimation in some underlying studies [15], [19], [24]. This approach causes an error that is difficult to quantify. It should, however, be small compared to most other errors given above.

Based on the considerable limitations described above, one may argue that analyses as those presented in this work are premature and should be postponed until better input data and especially 4-locus high-resolution HF data based on larger samples are available. As there are, however, substantial current donor recruitment efforts in an increasing number of countries in order to meet the needs of patients without HLA-identical family donor, this argument may be not appropriate.

Besides, key results of our analyses are sufficiently robust to be valid despite model and input data restrictions: First, we consistently found strong benefits of same-population donor recruitment for population-specific MP. In spite of the extensive current cross-border stem cell donor exchange, our data suggest that further substantial MP increases may be difficult to achieve without domestic donor recruitment for many populations. This observation is in accordance with previous results regarding Polish and German donor and patient populations [19]. We therefore recommend to critically re-assess national strategies that neglect domestic donor recruitment, especially if only few donors have been recruited so far. Second, though MP values depended considerably on sample sizes, the ranking of populations with respect to current MP and MP increases from future recruitment efforts remained relatively stable in scenarios that were based on different sample sizes. This observation suggests that our model is suited to identify populations with a specific need for donor recruitment efforts.

We concede, however, that it is doubtful if any prioritization of recruitment efforts by donor population that results from model calculations with the current input data limitations is more precise than a prioritization that is semi-quantitatively derived from basic parameters as, for example, current donor numbers or intra-population diversity. The results presented here should, therefore, be understood rather as a step to the enabling of a rational global stem cell donor recruitment strategy than as a final result. Though HF may also be estimated from incomplete data [23], [45], [46], it would be most helpful for the ongoing refinement of input data to type future new registrants “completely”, i.e., at least for the HLA loci A, B, C and DRB1 at high resolution. This strategy has also been identified as beneficial for donor registry operations [47]. Cost barriers to this approach should increasingly diminish due to the use of next-generation sequencing technologies for high-throughput donor HLA typing [48].

## Summary

Summarized, we extended established methods for MP estimation and built a model intended to estimate the need for and impact of stem cell donor recruitment efforts in various populations in a setting of international donor exchange. In spite of considerable limitations due to the nature of our input data, we could identify a need for domestic donor recruitment efforts for populations without large existing donor base as a general result. The availability of larger samples of completely typed donors will allow substantial refinements of the model calculations.

## Supporting Information

File S1 **Some mathematical properties of the scalar field** ***p_k_*** **.**
(DOC)

File S2 **Optimization of matching probabilities.**
(DOC)

File S3 **Matching probabilities in a 2-population scenario. MP** for Spanish patients (left) and German patients (right) from a registry including Spanish and German donors by registry size and composition. The current registry size and composition is depicted by the green dot.
(DOC)

File S4 **Donors needed to reach 0.8 MP.** Number of additional donors (compared to donor figures as given in Table 1) needed to reach 0.8 MP. Populations with current MP of >0.8 are not displayed.
(DOC)

File S5 **Results for 21 populations (including China).** Optimal recruitment of 5,000,000 donors with respect to maximization of the MP of a combined patient population including 21 populations.
(DOCX)

File S6 **Results for 20 populations (excluding China).** Optimal recruitment of 5,000,000 donors with respect to maximization of the MP of a combined patient population including 20 populations.
(DOCX)
