# Supplementary material for: Toward an Optimal Global Stem Cell Donor Recruitment Strategy
Source: PLoS One. 2014 Jan 30;9(1):e86605. doi: 10.1371/journal.pone.0086605 (PMC3907384; doi:10.1371/journal.pone.0086605)
Supplement: File S1 — Some mathematical properties of the scalar field pk . (DOC) [file pone.0086605.s001.doc]

**S1: Some mathematical properties of the scalar field *p_k_***

A point $P=\left(n_{1},n_{2},\ldots,n_{N}\right)$ of the *N*-dimensional space represents the registry with *n_i_* donors of population *i*. *n_i_* is always a natural number > 0. The function that describes the MP of patients of population *k* by registry size and composition can be pictured as *N*-dimensional hyper-surface *S_k_* = {(*n_1_,n_2_,…,n_N_*, *p_k_*(*n_1_,n_2_,…, n_N_*))} over *U_N_*. Points of *U_N_* that represent registries with identical matching probabilities (*p_k_* = const.) form an (*N*-1) - dimensional subset *U_N-_*_1_ of *U_N_*. Figures 1a and 1b show examples for *N* = 2. In this case the subsets of constant probability are one-dimensional contour lines.

In any subset *V_N_* of *U_N_* that is defined by a minimum and a maximum number of donors for each registry 1 < *n_i_,*_min_ < *n_i_* < *n_i_,*_max_ , *p_k_*(*n_1_,n_2_,…, n_N_*) does not have any local extrema and only one global maximum on its boundary. This can be seen as follows. First, $F_{p_{k}}$ does not have any critical point on *U_N_* because each partial derivative

$$\frac{\partial}{\partial n_{l}}F_{p_{k}}\left(n_{1},n_{2},\ldots,n_{N}\right)=-\sum_{i=1}^{M} f_{ik}\ln\left[1-f_{il}\right]\prod_{j=l}^{N} \left(1-f_{ij}\right)^{n_{j}}$$

is > 0 since one requires that there is at least one *i* with both $f_{ik}>0$ and $f_{il}>0$, indicating that the populations *k* and *l* share at least one common phenotype. Since, secondly, $F_{p_{k}}$ is a smooth function everywhere it follows as a direct consequence that $F_{p_{k}}$ has no extrema in *V_N_*. Hence, maxima of $F_{p_{k}}$ can only occur on the boundaries of *V_N_* where *n_i_* = *n_i_,*_min_ or *n_i_* = *n_i_,*_max_. On any boundary of *V_N_*, $F_{p_{k}}$ is given by $F_{p_{k}}\left(n_{1},n_{2},\ldots,n_{N}\right)$ with *n_i_* fixed and denoted by $F_{p_{k}}|_{i}$. The algebraic form of $F_{p_{k}}|_{i}$is the same as of $F_{p_{k}}$ (with one term less in the multiplication) and so within the boundary also no extrema exists but only on the boundary of the boundary. This applies until all parameters *n_i_* except one are kept fixed. The remaining one-dimensional function is always monotonic growing (as can be seen directly for the partial derivative of $F_{p_{k}}$) and thus has a maximum at *n_i_* = *n_i_,*_max_ . Consequently, the global maximum of $F_{p_{k}}$ is given by $F_{p_{k}}\left(n_{1,\text{max}},n_{2,\text{max}},\ldots,n_{N,\text{max}}\right)$.

As in the one-population case, the combined matching probability describes a scalar field over *U_N_* that also induces a hyper-surface. Since is just a linear function of several $F_{p_{k}}$, the same arguments about local and global maxima can be applied. As in the case of $F_{p_{k}}$, no first partial derivatives of vanishes and thus has no local maximum in any subset and  also features just one global maximum on its boundaries.
