# Supplementary material for: Toward an Optimal Global Stem Cell Donor Recruitment Strategy
Source: PLoS One. 2014 Jan 30;9(1):e86605. doi: 10.1371/journal.pone.0086605 (PMC3907384; doi:10.1371/journal.pone.0086605)
Supplement: File S2 — Optimization of matching probabilities. (DOC) [file pone.0086605.s002.doc]

**S2: Optimization of matching probabilities**

Matching probabilities can be optimized in two ways. First, one can determine the maximum matching probability for a given number of donors (case A) and, second, one can identify the minimum number of donors that is required to achieve a given matching probability (case B). For our studies, we have developed software that is capable to handle both cases and does this in a similar fashion.

As seen in Section S1, maxima of $F_{p_{k}}\left(n_{1},n_{2},\ldots,n_{N}\right)$ can only occur at the boundaries of a set *V_N_* that is given by 1 < *n_i_,*_min_ < *n_i_* < *n_i_,*_max_ and are defined by $F_{p_{k}}\left(n_{1,\text{max}},n_{2,\text{max}},\ldots,n_{N,\text{max}}\right)$. In all cases, *n_i_,*_min_ is readily given by current donor registry sizes. Therefore, for case A the question is how a given number of additional donors shall be distributed among the *N* different populations in a way that satisfies and gives the highest possible

$p_{k}=F_{p_{k}}\left(n_{1,\text{min}}+\Delta n_{1},n_{2,\text{min}}+\Delta n_{2},\ldots,n_{N,\text{min}}+\Delta n_{N}\right)$.

To accomplish this, we divide the total number of additional donors into sufficient small packages of donorssuch that . Note, that *j* does not count populations here but portions of the total amount of new donors . Each package is then added to each population separately to get new matching probabilities $\left(\hat{p}_{k}\right)_{1}=F_{p_{k}}\left(n_{1,\text{min}}+\delta n_{1},n_{2,\text{min}},\ldots,n_{N,\text{min}}\right)$ , $\left(\hat{p}_{k}\right)_{2}=F_{p_{k}}\left(n_{1,\text{min}},n_{2,\text{min}}+\delta n_{1},\ldots,n_{N,\text{min}}\right)$, and so forth. The package of donors is then attributed to the population with the highest matching probability and the other are discarded. Iteration of this procedure then yields the desired distribution of among the *N* populations.

Case B can be obtained in a similar way except that the total number of additional donors is not fixed at the beginning. Instead, the small packages are added to the populations until the desired matching probability is reached.

At this point it might be noteworthy to mention that a pure gradient method does not work here. The main reason for this is that distances between two points and in *U_N_* (and its subsets) are not measured by the common Euclidian norm

but by the 1-norm

.

This results from the fact that donors are discrete entities that belong to one of several distinct populations. It is, therefore, not possible to add, for example, 5 “mixed” donors to the registry instead of 3 donors from population *i* and 4 donors from population *j* (cf. Figure S2). It follows that donor recruitment can not be described by using “diagonal paths” within the space of donor numbers *U_N_*.

**Figure S2**

**
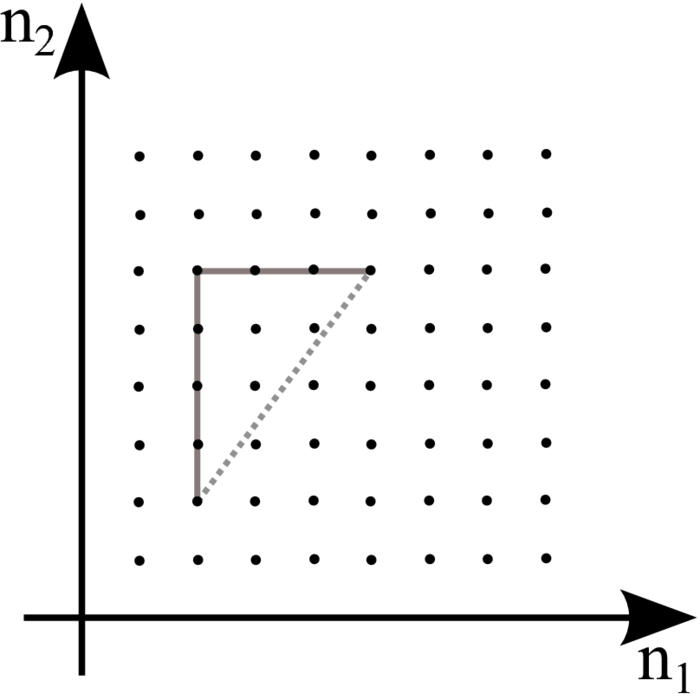
**

The impossibility of diagonal paths in donor recruitment. Each axis gives the number of donors in a distinct registry. Each point depicts a specific combination of registry sizes. Neighboring points differ by one donor. If one is to measure the amount of donors by which to arbitrary points differ, it has to be done in a donor-by-donor manner.
