# Supplementary figures and images for: Toward an Optimal Global Stem Cell Donor Recruitment Strategy
Source: PLoS One. 2014 Jan 30;9(1):e86605. doi: 10.1371/journal.pone.0086605 (PMC3907384; doi:10.1371/journal.pone.0086605)

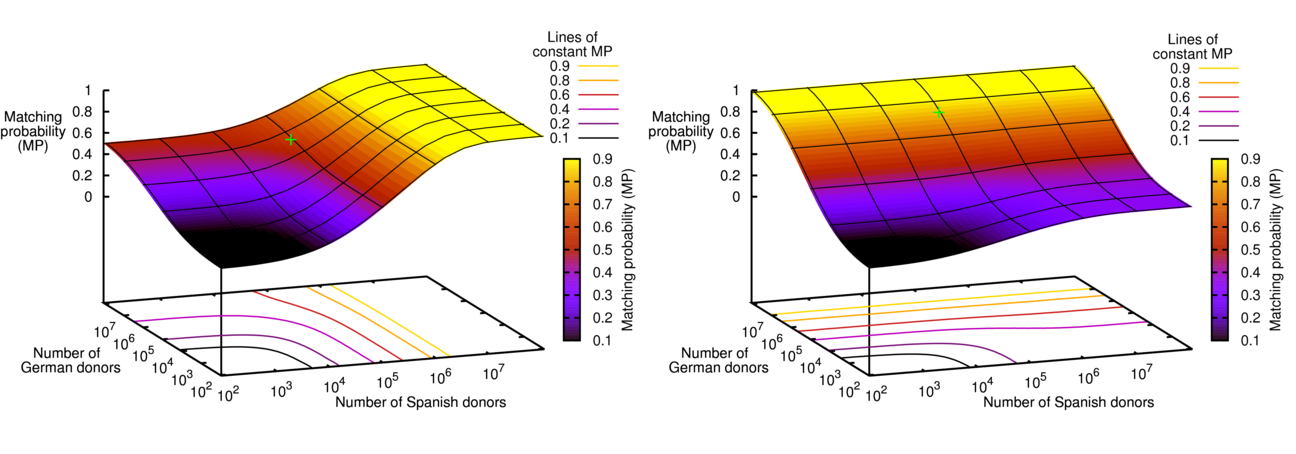

Supplement: File S3 — Matching probabilities in a 2-population scenario. MP for Spanish patients (left) and German patients (right) from a registry including Spanish and German donors by registry size and composition. The current registry size and composition is depicted by the green dot. (DOC) [file pone.0086605.s003.doc]

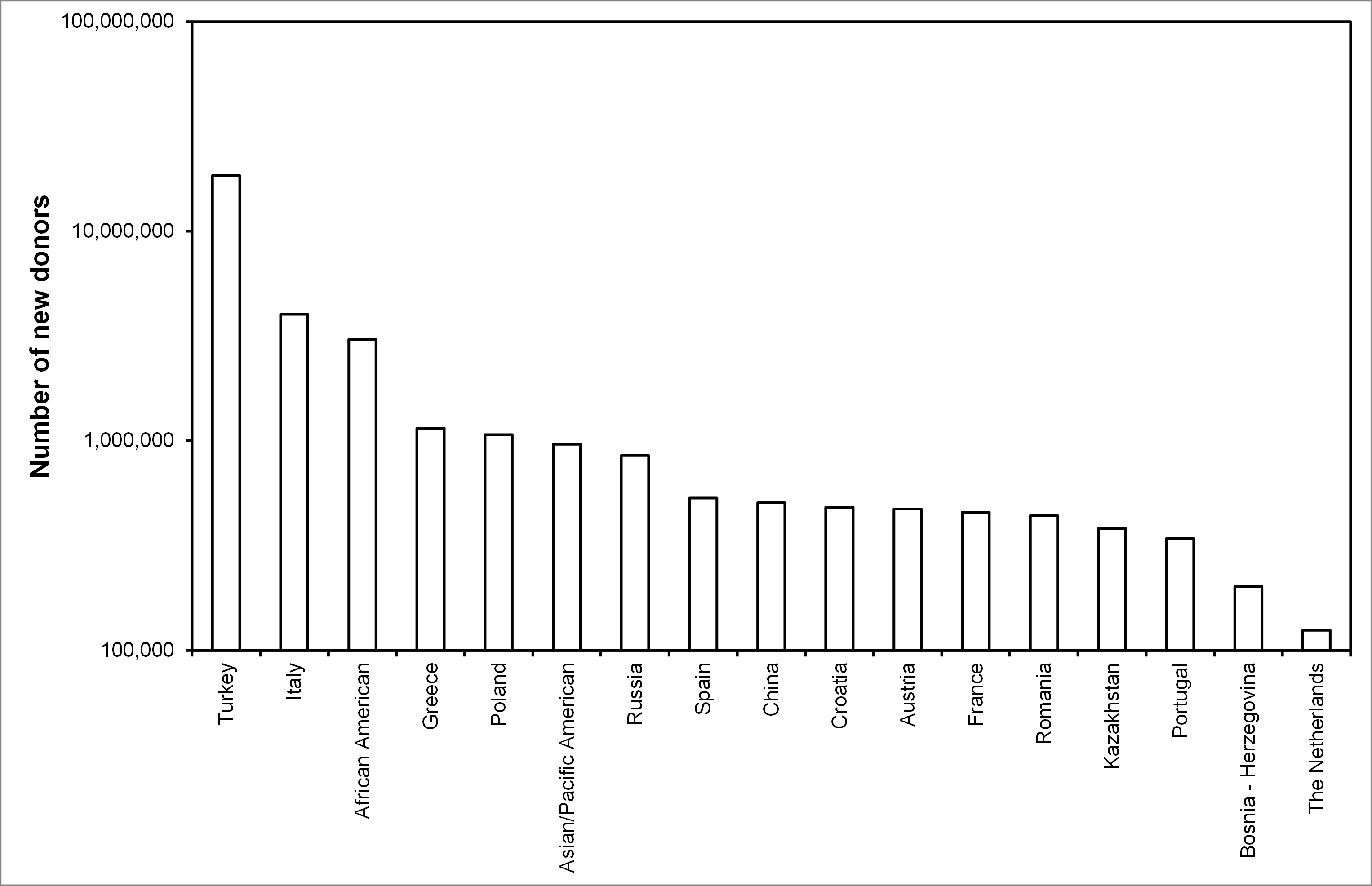

Supplement: File S4 — Donors needed to reach 0.8 MP. Number of additional donors (compared to donor figures as given in Table 1) needed to reach 0.8 MP. Populations with current MP of >0.8 are not displayed. (DOC) [file pone.0086605.s004.doc]
