# Supplementary material for: Toward an Optimal Global Stem Cell Donor Recruitment Strategy
Source: PLoS One. 2014 Jan 30;9(1):e86605. doi: 10.1371/journal.pone.0086605 (PMC3907384; doi:10.1371/journal.pone.0086605)
Supplement: File S5 — Results for 21 populations (including China). Optimal recruitment of 5,000,000 donors with respect to maximization of the MP of a combined patient population including 21 populations. (DOCX) [file pone.0086605.s005.docx]

**S5: Results for 21 populations (including China)**

|  | Current registry | | Donor recruitment | | | New registry | |
| --- | --- | --- | --- | --- | --- | --- | --- |
| Population | # of donors | MP | # of donors | % | Δ MP | # of donors | MP |
| African-American | 787,081 | 0.526 | 0 | 0 | 0.001 | 787,081 | 0.527 |
| Asian/Pacific-American | 769,846 | 0.683 | 0 | 0 | 0.008 | 769,846 | 0.691 |
| Austria | 66,014 | 0.698 | 0 | 0 | 0.010 | 66,014 | 0.708 |
| Bosnia-Herzegovina | 1,754 | 0.627 | 13,607 | 0.3 | 0.049 | 15,361 | 0.676 |
| China | 403,945 | 0.695 | 3,882,342 | 77.7 | 0.258 | 4,286,287 | 0.953 |
| Croatia | 30,823 | 0.648 | 0 | 0 | 0.022 | 30,823 | 0.670 |
| European-American | 3,690,624 | 0.905 | 0 | 0 | 0.003 | 3,690,624 | 0.908 |
| France | 200,172 | 0.712 | 128,088 | 2.7 | 0.038 | 328,260 | 0.750 |
| Germany | 4,343,558 | 0.852 | 0 | 0 | 0.004 | 4,343,558 | 0.856 |
| Greece | 37,760 | 0.494 | 32,025 | 0.6 | 0.054 | 69,785 | 0.548 |
| Hispanic-American | 1,080,082 | 0.865 | 0 | 0 | 0.002 | 1,080,082 | 0.867 |
| Italy | 345,265 | 0.579 | 0 | 0 | 0.016 | 345,265 | 0.595 |
| Kazakhstan | 2,833 | 0.679 | 22,409 | 0.5 | 0.034 | 25,242 | 0.713 |
| Poland | 297,464 | 0.747 | 0 | 0 | 0.013 | 297,464 | 0.760 |
| Portugal | 283,523 | 0.714 | 0 | 0 | 0.007 | 283,523 | 0.721 |
| Romania | 2,421 | 0.613 | 87,037 | 1.7 | 0.086 | 89,458 | 0.699 |
| Russia | 16,200 | 0.704 | 368,482 | 7.4 | 0.067 | 384,682 | 0.771 |
| Spain | 93,623 | 0.636 | 202,134 | 4.0 | 0.094 | 295,757 | 0.730 |
| The Netherlands | 42,733 | 0.755 | 0 | 0 | 0.009 | 42,733 | 0.764 |
| Turkey | 114,248 | 0.379 | 263,881 | 5.3 | 0.066 | 378,129 | 0.445 |
| United Kingdom | 815,660 | 0.895 | 0 | 0 | 0.001 | 815,660 | 0.896 |
| Combined | 13,425,629 | 0.711 | 5,000,000 | 100 | 0.162 | 18,425,634 | 0.873 |

Optimal recruitment of 5,000,000 donors with respect to maximization of the MP of a combined patient population including 21 populations.
