# Supplementary material for: Toward an Optimal Global Stem Cell Donor Recruitment Strategy
Source: PLoS One. 2014 Jan 30;9(1):e86605. doi: 10.1371/journal.pone.0086605 (PMC3907384; doi:10.1371/journal.pone.0086605)
Supplement: File S6 — Results for 20 populations (excluding China). Optimal recruitment of 5,000,000 donors with respect to maximization of the MP of a combined patient population including 20 populations. (DOCX) [file pone.0086605.s006.docx]

**S6: Results for 20 populations (excluding China)**

|  | Current registry | | Donor recruitment | | | New registry | |
| --- | --- | --- | --- | --- | --- | --- | --- |
| Population | # of donors | MP | # of donors | % | Δ MP | # of donors | MP |
| African-American | 787,081 | 0.526 | 210,996 | 4.2 | 0.044 | 998,077 | 0.570 |
| Asian/Pacific-American | 769,846 | 0.677 | 0 | 0.0 | 0.003 | 769,846 | 0.680 |
| Austria | 66,014 | 0.697 | 0 | 0.0 | 0.030 | 66,014 | 0.727 |
| Bosnia-Herzegovina | 1,754 | 0.626 | 57,472 | 1.6 | 0.124 | 59,226 | 0.750 |
| Croatia | 30,823 | 0.648 | 0 | 0.0 | 0.057 | 30,823 | 0.705 |
| European-American | 3,690,624 | 0.905 | 0 | 0.0 | 0.009 | 3,690,624 | 0.914 |
| France | 200,172 | 0.678 | 692,804 | 13.7 | 0.104 | 892,976 | 0.815 |
| Germany | 4,343,558 | 0.852 | 0 | 0 | 0.012 | 4,343,558 | 0.864 |
| Greece | 37,760 | 0.493 | 206,238 | 4.1 | 0.170 | 243,998 | 0.663 |
| Hispanic-American | 1,080,082 | 0.865 | 0 | 0.0 | 0.004 | 1,080,082 | 0.869 |
| Italy | 345,265 | 0.579 | 583,601 | 11.7 | 0.099 | 928,866 | 0.678 |
| Kazakhstan | 2,833 | 0.678 | 124,445 | 2.5 | 0.092 | 127,278 | 0.770 |
| Poland | 297,464 | 0.746 | 0 | 0.0 | 0.035 | 297,464 | 0.781 |
| Portugal | 283,523 | 0.714 | 0 | 0.0 | 0.019 | 283,523 | 0.733 |
| Romania | 2,421 | 0.612 | 311,612 | 6.2 | 0.180 | 314,033 | 0.792 |
| Russia | 16,200 | 0.703 | 1,103,536 | 22.1 | 0.123 | 1,119,736 | 0.826 |
| Spain | 93,623 | 0.636 | 662,283 | 13.6 | 0.191 | 755,906 | 0.827 |
| The Netherlands | 42,733 | 0.755 | 61,477 | 1.2 | 0.045 | 104,210 | 0.800 |
| Turkey | 114,248 | 0.377 | 985,539 | 19.7 | 0.161 | 1,099,787 | 0.538 |
| United Kingdom | 815,660 | 0.895 | 0 | 0.0 | 0.005 | 815,660 | 0.900 |
| Combined | 13,425,629 | 0.732 | 5,000,000 | 100% | 0.071 | 18.425,629 | 0.803 |

Optimal recruitment of 5,000,000 donors with respect to maximization of the MP of a combined patient population including 20 populations.
